# Telemedicine Hybrid Care Models in Gastroenterology Outpatient Care: Results from a German Tertiary Center

**DOI:** 10.3390/jcm14072471

**Published:** 2025-04-04

**Authors:** Nada Abedin, Christian Kilbinger, Alexander Queck, Nina Weiler, Anita Pathil, Ulrike Mihm, Christoph Welsch, Irina Blumenstein, Alica Kubesch-Grün, Stefan Zeuzem, Georg Dultz

**Affiliations:** Medical Clinic 1, University Hospital, Goethe University Frankfurt, 60596 Frankfurt am Main, Germany

**Keywords:** telemedicine, hybrid care, pandemic

## Abstract

**Background:** With the COVID-19 pandemic, a rapid adoption of telemedicine became necessary. Data regarding its implementation in specialized hepatology/IBD care remain limited. This study evaluated telemedicine’s effectiveness and safety during the pandemic at a German tertiary center and explored its integration into future hybrid care models. **Methods:** In a retrospective study, we analyzed 3147 patient encounters at the outpatient clinic of the Department for Gastroenterology and Hepatology at the University Hospital Frankfurt between March and June 2020. We assessed patient characteristics, appointment adherence, and outcomes across the three specialized clinics: hepatology (n = 1963), liver transplant (n = 594), and IBD (n = 590). Multivariate regression analysis identified predictors of successful telemedicine utilization. **Results:** Out of all appointments, 1112 (35.3%) were conducted via telemedicine, with significantly different adoption rates across clinics (hepatology, 40.4%; liver transplant, 32.8%; IBD, 21.0%, *p* < 0.01). Adherence rates were comparable between telemedicine (91.3%) and in-person visits (90.5%). Multivariate analysis identified age (OR 1.009, 95%CI 1.004–1.014, *p* < 0.001), metabolic-associated steatotic liver disease (OR 1.737, 95%CI 1.400–2.155, *p* < 0.001), and post-liver transplant status (OR 1.281, 95%CI 1.001–1.641, *p* = 0.049) as independent predictors of successful telemedicine utilization. HBV/HDV coinfection (OR 0.370, 95%CI 0.192–0.711, *p* = 0.003) and required endoscopy (OR 0.464, 95%CI 0.342–0.630, *p* < 0.001) were associated with in-person care. Hospitalization rates were low and comparable across modalities, confirming telemedicine’s safety. **Conclusions:** This study demonstrates that telemedicine can be successfully implemented in specialized gastroenterology and hepatology care, with high compliance rates comparable to in-person visits. Patient characteristics and disease-specific factors influence the suitability for telemedicine, supporting a stratified approach to hybrid care models, which can optimize resource utilization while maintaining quality of care. Particularly stable MASLD patients, well-controlled post-transplant recipients beyond one year, and IBD patients in sustained remission can be properly managed through telemedicine with annual in-person assessments.

## 1. Introduction

The COVID-19 pandemic caused a disruption in healthcare delivery and clinical care worldwide [1,2]. Healthcare providers needed to rapidly adapt their care models to maintain essential medical services while minimizing infection risks. This adaptation was particularly challenging in specialized fields such as gastroenterology and hepatology, which serve highly infection-susceptible patients with chronic conditions who require consistent monitoring and care continuity [3].

Despite advances in technology and growing evidence of its benefits, telemedicine was infrequently used in gastroenterology and hepatology before the pandemic [4,5]. In Germany, regulatory restrictions, including the “Fernbehandlungsverbot” (remote treatment prohibition), had historically constrained telemedicine implementation [6,7]. With the pandemic, these regulations needed to be rapidly addressed, and policy changes were induced to enable broader telemedicine utilization [8,9,10].

Because of its very diverse patient population, specialized gastroenterology care presents unique challenges for telemedicine implementation. Patients with liver disease, particularly those with advanced liver disease such as liver cirrhosis, require regular monitoring of disease progression and complications. Liver transplant recipients need consistent immunosuppression management and surveillance for complications [11,12,13]. Inflammatory bowel disease (IBD) patients often present with ongoing symptoms, require frequent adjustments in their therapeutic regimens based on disease activity, and/or have to present in person to receive intravenous therapy [4,14]. Each of these patient groups presents distinct considerations for remote care feasibility.

The existing literature on telemedicine in gastroenterology during the COVID-19 pandemic has primarily focused on general gastroenterology practices or single disease entities [8,12,15,16,17]. There have been few studies on the implementation of telehealth across various gastroenterology subspecialties to evaluate its suitability for particular diseases. Furthermore, while several studies have reported on the technical feasibility of telemedicine, fewer have examined patient characteristics and disease-specific factors that might influence the success of remote care delivery. In this study, successful telemedicine utilization was defined as technically complete remote consultations providing adequate clinical management, without subsequent adverse outcomes or the immediate need for in-person follow-up, thus encompassing both technical completion and clinical suitability. As the demand for specialized care grows and becomes increasingly centralized, hybrid care models will become an essential part of care delivery. Recent meta-analyses have shown comparable outcomes between telemedicine and in-person care models in chronic disease management, yet specialized gastroenterology populations such as patients with chronic hepatitis and liver cirrhosis or post-transplant patients remain underrepresented [18,19,20,21].

Our study addresses this gap by analyzing a large cohort of patients from three specialized gastroenterology clinics during the early pandemic period when the lockdown had just started and flexible care solutions were urgently needed. We aimed to evaluate not only the feasibility of telemedicine implementation but also to identify patient characteristics and disease-specific factors that predict successful remote care delivery. This information is crucial for developing evidence-based strategies for hybrid care models that can optimize healthcare delivery in the future.

## 2. Materials and Methods

### 2.1. Study Design and Population

In a retrospective analysis, we analyzed all patient encounters at the Department for Gastroenterology and Hepatology at the University Hospital Frankfurt, Germany, between 2 March 2020 and 10 June 2020, the early pandemic period. This timeframe represents the critical initial phase of the pandemic, when strict lockdown measures required rapid adaptation of healthcare delivery models, and allowed us to assess the feasibility of telemedical care with limited resources. The study included all scheduled appointments in three specialized clinics: hepatology, liver transplantation (LTX), and inflammatory bowel disease (IBD).

### 2.2. Data Collection

Patient data were extracted from the hospital’s electronic medical record system (ORBIS). Each appointment was categorized as either in-person or telemedicine consultation. Comprehensive demographic data, including age, gender, country of origin, and employment status, were collected from the patients’ files. Clinical parameters included primary diagnosis, comorbidities, medication regimens, laboratory values, the presence of multidrug-resistant organisms (MDRs), and follow-up data. For liver disease patients, Model for End-Stage Liver Disease (MELD) scores were calculated using the following formula: MELD = 9.57 × ln(creatinine, in mg/dL) + 3.78 × ln(bilirubin in mg/dL) + 11.20 × ln(INR) + 6.43 [22]. Additionally, all diagnostic procedures performed during the study period, including endoscopy and laboratory testing, were documented.

### 2.3. Telemedicine Implementation

**Figure 1:**
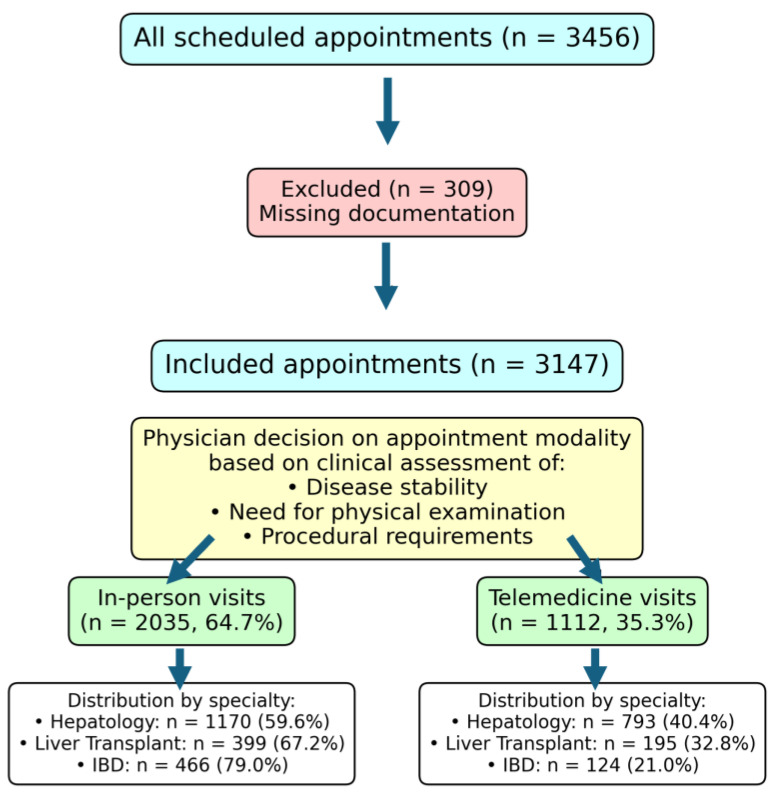
Flow diagram showing the selection process from initial appointments through physician determination of visit modality. Appointment modality was based on clinical assessment.

Appointment modalities were determined by treating physicians for each patient individually, based on three key criteria, (1) clinical stability and disease severity, (2) the need for physical examination or procedures, and (3) medication complexity, ensuring patient-centered and safe care delivery (Figure 1). Telemedicine consultations were conducted via telephone, as this represented the most widely accessible technology for the patient population during the early pandemic period. Barring exceptional circumstances, all first-time consultations were scheduled as in-person visits to ensure clinical assessment was possible.

### 2.4. Outcome Measures

Primary outcomes were appointment compliance rates for both in-person and telemedicine visits. Secondary outcomes included factors associated with successful telemedicine utilization and identification of patient subgroups most suitable for remote care. Appointment compliance was defined as attendance at scheduled in-person visits or successful telephone contact for telemedicine appointments.

### 2.5. Statistical Analysis

Statistical analyses were performed using STATA version 17.0 and SPSS version 28.0. Descriptive statistics were calculated for demographic and clinical characteristics. For a comparison of compliance rates between in-person and telemedical care, the chi-square test was used. Binary logistic regression analyses were performed to identify predictors of successful telemedicine utilization. Variables showing significance (*p* < 0.05) in univariate analysis were included in multivariate models. The results are presented as odds ratios (ORs) with 95% confidence intervals (CIs).

For the regression analyses, two models were developed: one examining factors associated with general appointment compliance (regardless of visit type) and another investigating predictors of successful telemedicine utilization versus in-person care requirements. Variables were selected based on clinical relevance and previously published data. All statistical tests were two-sided, with *p* < 0.05 considered statistically significant.

### 2.6. Ethics Committee’s Approval

The study was approved by the institutional review board of Frankfurt University Hospital (Ethikkommission der Goethe-Universität Frankfurt, decision 20-692, decision date, 30 April 2020).

## 3. Results

### 3.1. Demographics and Baseline Characteristics

After excluding 309 entries with missing documentation, we included 3147 patient encounters from 3456 scheduled appointments between 2 March and 10 June 2020. Baseline characteristics are included in Table 1. The included entries comprised 1963 hepatology (62.4%), 594 liver transplant (18.9%), and 590 IBD (18.7%) clinic visits. The median age was 51 years (range, 15–87), with liver transplant patients being the oldest (median, 58 years), followed by hepatology (52 years) and IBD patients (42 years). Male patients comprised 51.6% of the total population, with the highest proportion in the liver transplant clinic (61.5%), followed by hepatology (50.1%) and IBD (47.0%).

Most patients were of German origin (78.9%), followed by other European countries (9.0%), Asia (3.9%), and Africa (1.1%); the origin of 7.0% was either different or unknown. Language barriers were documented in 2.7% of encounters, most frequently in hepatology (3.8%) compared to liver transplant (1.4%) and IBD (0.5%). Despite restrictive hospital policies, 2.2% of encounters required an accompanying person, most commonly in the liver transplant clinic (3.4%), followed by hepatology (2.3%) and IBD clinics (0.3%).

### 3.2. Disease Distribution and Medical Management

In the hepatology clinic, chronic hepatitis B (30%), metabolic-associated fatty liver disease (MASLD, 18.6%), and chronic hepatitis C (13.7%) were most common. Autoimmune hepatitis and primary biliary cholangitis/primary sclerosing cholangitis (PBC/PSC) comprised 6.1% and 7.9%, respectively. Cirrhosis was documented in 19.3% of all included cases. The IBD clinic primarily managed Crohn’s disease (47.3%) and ulcerative colitis (38.8%). In the liver transplant clinic, 52.7% were post-transplant patients, and 47.3% were on the waiting list, with alcohol-associated liver disease being the most common underlying cause (19.4%), followed by chronic HCV infection (15.7%), chronic HBV infection (10.3%), and MASLD (10.4%).

The median number of prescribed medications varied significantly across specialties, being highest in liver transplant patients (median, seven medications) compared to IBD and hepatology patients (median, three medications each). Immunosuppressive therapy was most frequent in liver transplant visits (57.2%), followed by IBD (38.3%) and hepatology (10.7%). Laboratory testing was performed in 50.4% of all encounters, with the highest rate in IBD patients (61.0%), followed by liver transplant (53.5%) and hepatology patients (46.3%).

### 3.3. Appointment Distribution and Follow-Up Patterns

**Figure 2:**
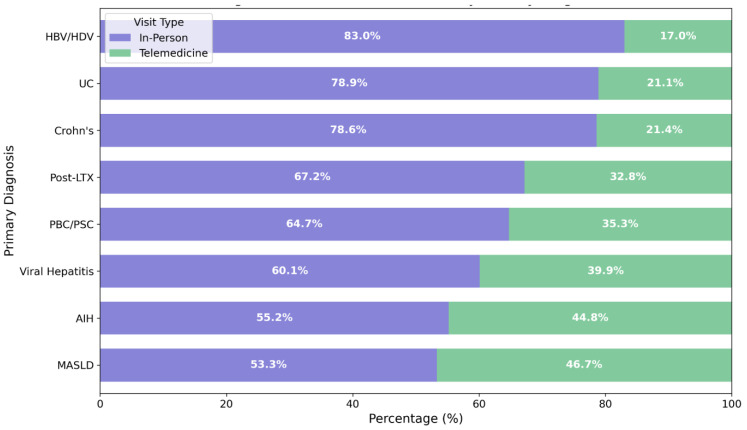
Telemedicine utilization by primary diagnosis. Abbreviations: MASLD: metabolic-associated steatotic liver disease, AIH: autoimmune hepatitis, PBC/PSC: primary biliary/primary sclerosing cholangitis, LTX: liver transplant, UC: ulcerative colitis, and HBV/HDV: hepatitis B/D virus coinfection.

The appointment distribution is shown in Table 2 and Figure 2 for the different disease entities. Out of all appointments, a total of 1112 (35.3%) could be performed via telemedicine, which left only 64.7% (2035) coming to the hospital. The IBD clinic had the highest proportion of on-site visits (79%), reflecting frequent intravenous infusions (n = 208, 35%) and clinical evaluation needs (n = 300, 50.9%). By contrast, hepatology utilized more phone calls (40.4%), driven by stable chronic conditions requiring no immediate intervention. The liver transplant clinic used phone appointments for 32.8% of visits.

Follow-up intervals differed significantly across specialties with a median of 95 days between visits for hepatology, 52 days for IBD, and 42 days for liver transplant clinics. Endoscopic procedures were required in 8.8% of the total encounters, most frequently in liver transplant (18.4%) compared to hepatology (5.6%). In these cases, in-person follow-up was significantly more common (79.1% vs 20.9%, *p* < 0.001).

### 3.4. Appointment Compliance and Predictors of Success

**Figure 3:**
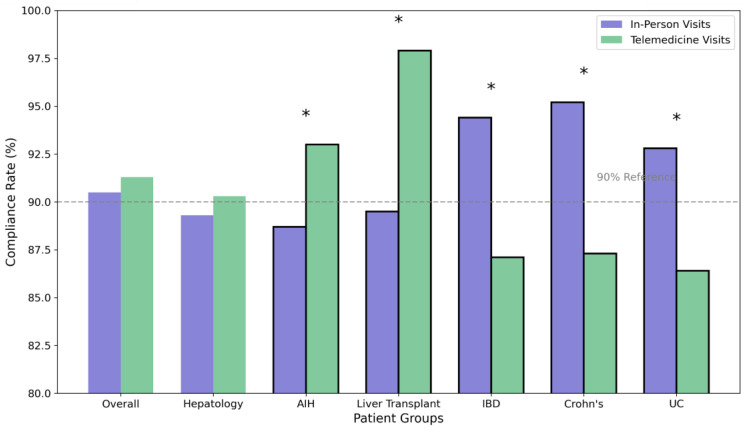
Appointment compliance rates by specialty and visit type. Abbreviations: AIH: autoimmune hepatitis, IBD: inflammatory bowel disease, UC: ulcerative colitis. * Indicates statistically significant differences (*p* < 0.05) between in-person and telemedicine compliance rates.

Overall compliance rates, as shown in Table 3 and visualized in Figure 3, were comparable (*p* = 0.428) between in-person (90.5%) and telemedicine visits (91.3%). The liver transplant clinic demonstrated the highest telemedicine compliance rate (97.9% vs. 89.5 in-person, *p* < 0.001), followed by hepatology (90.3% vs. 89.3 in-person, *p* = 0.512) and IBD (87.1% vs. 94.4 in-person, *p* = 0.024). Within the hepatology cohort, compliance rates for patients with AIH were 93% for telemedical care vs. 88.7% for in-person visits, *p* = 0.042. There were no significant differences in compliance rates for the other subgroups in the hepatology cohort (viral hepatitis, *p* = 0.124; PBC/PSC, *p* = 0.682; MASLD, *p* = 0.724). Within the IBD subgroup, compliance rates were significantly higher in the in-person care group (Crohn’s disease, *p* = 0.018; ulcerative colitis, *p* = 0.032).

Predictors of remote telemedical care were analyzed using binary regression analysis (Table 4). In univariate analysis, both the IBD clinic and liver transplant clinic showed higher odds of successful appointment completion compared to the hepatology clinic. Stable disease, reflected by longer intervals since the previous visit, was also associated with telemedical care. Older age was identified as a significant factor for appointment compliance. Disease-specific predictors significantly associated with telemedical care in the univariate analysis included chronic HCV, AIH, MASLD, and patients with post-transplant status.

In the following multivariate analysis, stable disease, as indicated by longer intervals, remained significantly associated with telemedical care, as well as MASLD and post-transplant status.

Conversely, Crohn’s disease and ulcerative colitis were associated with in-person visits to the IBD clinic in the univariate analysis. In hepatology, HBV/HDV coinfection, a need for endoscopy, and first-time consultations were associated with in-person care. HBV/HDV and a need for endoscopy remained significantly associated with in-person visits in the multivariate analysis.

### 3.5. Clinical Outcomes

During the study period and one-year follow-up, no significant differences were observed in emergency department visits or hospital admissions between patients managed via telemedicine versus in-person care.

## 4. Discussion

This study provides comprehensive insights into telemedicine implementation in specialized gastroenterology care during the early COVID-19 pandemic. The results demonstrate both the feasibility and limitations of remote care delivery across different patient populations and reveal key patterns to inform sustainable hybrid care models in gastroenterology.

The overall high compliance rates for both telemedicine (91.3%) and in-person visits (90.5%) challenge the concern that remote care might lead to reduced patient engagement or worse care delivery. This finding is particularly significant given the study’s timing during the initial pandemic phase when healthcare systems worldwide reported substantial decreases in routine care utilization [15,23,24]. The comparable compliance rates suggest that appropriately selected patients—in our study, a total of 1112 patients (35.3%)—can be effectively managed through telemedicine platforms [25].

Telemedicine utilization varied across specialties, reflecting inherent differences in key requirements. The higher rate of telemedicine adoption in hepatology (40.4%) compared to IBD (21.0%) likely reflects the nature of disease monitoring in these populations. Patients with MASLD showed the highest telemedicine suitability (OR 1.333, *p* < 0.05), making them ideal candidates for telemedicine-based follow-up care. Our findings align with recent studies demonstrating the successful remote management of metabolic liver disease [5,15,26]. The high success rate in this population can be attributed to several factors: the relatively stable nature of the condition, the importance of lifestyle modifications in management, and the predictable intervals for laboratory monitoring. This suggests that MASLD patients might be ideal candidates for primarily telemedicine-based follow-up with periodic in-person assessments [27].

Patients with autoimmune hepatitis also demonstrated higher telehealth utilization (44.8%) compared to other hepatology patients, with univariate analysis confirming AIH as a predictor of successful telehealth utilization (OR 1.591, *p* < 0.01). However, AIH did not remain significantly associated with telemedical care in the multivariate model, which reflects the need for regular laboratory monitoring and therapy adjustments. Given the high infection risk of patients undergoing immunosuppressive therapy during the pandemic, it was vital for these patients to thoroughly assess the necessity of an in-person presentation. As recent studies have suggested, stable AIH patients can be effectively monitored through remote care protocols [28], particularly when following established immunosuppression regimens, keeping in mind the importance of a flexible hybrid care approach due to possible disease flares.

The liver transplant population presents a more complex picture. While showing the highest compliance with telemedicine appointments (97.9%), these patients required a balanced approach between remote and in-person care. The success of telemedicine in this historically high-risk population challenges traditional post-transplant monitoring protocols. However, the need for regular laboratory monitoring and physical examination necessitated maintaining a significant proportion of in-person visits (67.2%). This finding suggests that a hybrid care model might be optimal for transplant recipients, with telemedicine visits interspersed between necessary in-person evaluations [15].

The association between older age and successful telemedicine utilization challenges the common perception that elderly patients might struggle with remote care modalities. This finding may reflect both the simplicity of phone-based consultations and the greater motivation for healthcare engagement among older patients, as documented in previous studies [13,29,30].

Identifying specific factors that necessitate in-person care, like HBV/HDV coinfection and endoscopy requirements, provides practical guidance for risk stratification [31,32,33]. These findings suggest that certain patient subgroups require more careful consideration before the implementation of remote care strategies.

The impact of medication burden on care delivery presents an interesting paradox. Despite the higher medication burden on liver transplant patients (median, seven medications), this did not negatively impact telemedicine success. This suggests that medication complexity alone should not be considered a barrier to telemedicine implementation [13,34].

The lower telemedicine utilization in IBD care (21.0%) reflects the unique challenges in managing these patients remotely. The need for physical examination, endoscopic evaluation, and the regular administration of biological therapies necessitates more frequent in-person contact. However, the high compliance rates even in this group suggest that select IBD patients, particularly those in stable remission, might benefit from periodic telemedicine follow-up [30,35].

The comparable compliance rates between telemedicine and in-person visits suggest that remote care options might improve overall healthcare access. The median follow-up intervals (hepatology: 95 days; LTX: 42 days; IBD: 52 days) provide a benchmark for planning hybrid care schedules. The longer intervals associated with successful telemedicine utilization suggest that stable patients can be safely monitored remotely with extended intervals between in-person assessments.

The role of laboratory monitoring emerged as a crucial consideration, with significant variations across specialties. This highlights the need for coordinated care planning that integrates remote consultations with necessary in-person testing.

Overall, our findings have important implications for healthcare system design and align with and expand on recent studies. The high success rates of telemedicine suggest that hybrid care models could optimize resource utilization while maintaining quality of care. This is particularly relevant given the increasing burden of chronic liver disease and IBD globally, combined with limited specialist availability in many regions. In a recent study, patients and providers approved of telemedical care in regular medical care [20,36].

The differential success rates across conditions and patient populations emphasize the need for flexible, patient-centered care models rather than one-size-fits-all approaches to telemedicine implementation. This suggests that gastroenterology practices should develop clear protocols for patient stratification and care pathway selection.

Additionally, the economic implications of telemedicine use need more detailed consideration beyond the clinical outcomes in our study. For specialized gastroenterology care, where patients often travel far distances to specialized centers, telemedicine could significantly reduce patient-incurred expenses, including transportation costs and accommodation needs. From a provider perspective, a differentiated resource allocation could address the growing demand–supply gap in these specialized services. However, initial investments are needed initially to ensure secure technological infrastructure, provider training, and workflow redesign. Future cost-effectiveness analyses should comprehensively evaluate both direct treatment costs and indirect economic benefits, particularly focusing on disease-specific outcomes that might influence further long-term healthcare utilization. With the increasing worldwide shortage of specialists, hybrid care models become increasingly relevant.

Our findings support the development of stratified hybrid care models in gastroenterology, with several key recommendations for clinical practice. We propose a three-tier system for patient categorization in specialized gastroenterology care:

1. Telemedicine-preferred: Stable MASLD patients, well-controlled post-transplant recipients beyond one year, and IBD patients in sustained remission. These patients can be primarily managed through telemedicine with annual in-person assessments.
2. Hybrid care: Patients requiring regular monitoring but showing stable disease patterns. This includes most liver transplant recipients, compensated cirrhosis patients, and IBD/AIH/PBC patients on stable therapy. A structured alternating schedule of in-person and telemedicine visits appears optimal.
3. In-person priority: Patients with HBV/HDV coinfection, decompensated cirrhosis, active IBD flares, or those requiring frequent procedural interventions should maintain primarily in-person care with telemedicine serving as supplementary support.

The high compliance rates across all modalities suggest that this stratified approach could optimize resource utilization while maintaining care quality. Implementation should consider local resources, patient preferences, and specific disease characteristics. While the proposed three-tier model is designed to categorize patients based on disease severity and telemedicine suitability, it is important to note that patients may transition between tiers depending on their clinical status, necessitating regular re-evaluation of care pathways.

Regarding practical implementation, several challenges need consideration, including different levels of digital literacy and technological access among patient populations, as has been discussed by Hakak et al. [20]. Our findings challenge previous assumptions about age-related barriers, but other factors such as socioeconomic status might influence telemedicine accessibility. Concerning providers, readiness differs across healthcare settings, and reimbursement policies need to be consistent to ensure sustainability. Finally, appropriate patient stratification should be supported by clinical decision-support tools and robust communication systems [37].

The presented study has several limitations that should be acknowledged when interpreting the results. First, the single-center design may limit generalizability to other healthcare settings, particularly those with different infrastructure, patient populations, or regulatory frameworks. While the large sample size and diverse patient population in our study mitigate this concern, multi-center studies across different healthcare systems would provide more evidence. Second, the study period represents the early pandemic phase, when both healthcare providers and patients were adapting to new care delivery models; hence, long-term outcomes and sustained adoption patterns are not captured in our study. Third, the telemedicine implementation in this hospital was limited to telephone consultations and may not fully reflect the potential benefits of video-based consultations [10,30]. Additionally, data on patient satisfaction were not collected, which could provide insights into the acceptability and perceived quality of telemedicine services. Furthermore, while we assessed basic safety outcomes like emergency department visits and hospitalizations, more nuanced quality metrics specific to each disease entity were not systematically evaluated. Despite these limitations, our findings provide important insights into the feasibility and safety of telemedicine in specialized gastroenterology care during a period of significant healthcare disruption.

Several key areas warrant further investigation to optimize hybrid care models in gastroenterology, such as the assessment of long-term outcomes with prospective studies comparing clinical outcomes between traditional and hybrid care models, particularly focusing on disease-specific endpoints such as sustained viral response in hepatitis, graft survival in transplant recipients, and remission rates in IBD. Furthermore, comprehensive economic analysis should evaluate the direct impact of hybrid care models, including healthcare system costs, patient-incurred expenses, and potential savings from reduced hospital visits. Thirdly, technology evaluation should explore optimal platforms beyond telephone consultations, including secure video conferencing, remote monitoring devices, and patient-facing applications that enhance engagement and self-management. Finally, both technology integration and quality metrics need to be pushed to further develop and validate optimal care delivery. These should include standardized quality metrics and patient-reported outcome measures specific to telemedicine in gastroenterology. The integration of artificial intelligence and clinical decision support tools for appropriate patient stratification should be regularly discussed.

To summarize, our study demonstrates that telemedicine can be successfully integrated into specialized gastroenterology care, with high compliance rates comparable to traditional in-person care. The identification of specific predictors for telemedicine success provides a framework for patient stratification and care pathway optimization. Our study showed that patients with stable disease (MASLD, post-transplant) and those in remission (IBD) are great candidates for a hybrid care approach. As healthcare systems evolve beyond the pandemic era, these findings support the development of sustainable hybrid care models that can enhance healthcare access while maintaining quality of care.

The transition to hybrid care models represents not merely a response to pandemic conditions but an opportunity to reshape specialized gastroenterology care delivery for improved accessibility and efficiency. The continued evaluation and refinement of these models will be essential to optimizing patient outcomes in the evolving healthcare landscape.

## 5. Conclusions

In conclusion, this study demonstrates that telemedicine can be successfully integrated into specialized gastroenterology care, with high compliance rates comparable to traditional in-person care. The identification of specific predictors for telemedicine success—including stable MASLD, well-controlled post-transplant status, and IBD in sustained remission—provides a framework for patient stratification and care pathway optimization. Our findings challenge assumptions about digital barriers in elderly patients, showing that age was not associated with lower adherence rates or telemedicine utilization. The comparable safety outcomes between modalities support the development of sustainable, disease-specific hybrid care models that can enhance healthcare access while maintaining quality of care. With appropriate patient selection and strategic implementation, telemedicine represents not merely a pandemic-necessitated adaptation but an opportunity to transform specialized gastroenterology care delivery for improved accessibility, efficiency, and patient-centeredness in the evolving healthcare landscape.

Beyond individual patient care, our findings have broader implications for healthcare system design and policy development. The success of telemedicine across diverse patient populations in our study supports policies that institutionalize hybrid care models as standard practice rather than emergency alternatives. Regulatory frameworks should evolve to facilitate appropriate reimbursement parity, cross-border licensure for specialized care, and technology standards that ensure equitable access. Healthcare systems should consider redesigning clinical workflows to incorporate the stratified care model identified in our study, potentially reducing costs while optimizing specialist resource allocation. Future implementation research should focus on developing standardized protocols for risk stratification in gastroenterology subspecialties to guide appropriate modality selection and care pathways.

## Figures and Tables

**Table 1:** Baseline characteristics across specialties.

Characteristic	Total (n = 3147)	Hepatology (n = 1963)	Liver Transplant (n = 594)	IBD (n = 590)
Age, median (range)	51 (15–87)	52 (15–87)	58 (15–87)	42 (18–86)
Male gender, n (%)	1625 (51.6)	983 (50.1)	365 (61.5)	277 (47.0)
Primary diagnosis, n (%)				
MASLD	433 (13.8)	366 (18.6)	62 (10.4)	5 (0.9)
Viral hepatitis	1031 (32.8)	857 (43.7)	154 (25.9)	20 (3.4)
AIH	192 (6.10)	137 (6.98)	43 (7.24)	12 (2.03)
Crohn’s disease	294 (9.34)	15 (0.76)	0	279 (47.29)
Ulcerative colitis	280 (8.90)	19 (0.97)	32 (5.39)	229 (38.81)
Other	1109 (35.2)	706 (36.0)	346 (58.3)	57 (9.6)
Medications				
Median number (range)	3 (0–19)	2 (0–17)	7 (0–19)	3 (0–19)
Immunosuppression, n (%)	776 (24.7)	210 (10.7)	340 (57.2)	226 (38.3)
Clinical parameters				
MDR organisms present, n (%)	282 (9.0)	70 (3.6)	182 (30.6)	30 (5.1)
Required endoscopy, n (%)	278 (8.8)	110 (5.6)	109 (18.4)	59 (10.0)
Comorbidities				
Arterial hypertension, n (%)	685 (21.77)	420 (21.40)	204 (34.34)	61 (10.34)
Atrial fibrillation, n (%)	69 (2.19)	38 (1.94)	28 (4.71)	3 (0.51)
Renal insufficiency, n (%)	192 (6.10)	53 (2.70)	135 (22.73)	4 (0.68)
Diabetes, n (%)	451 (14.33)	268 (13.65)	152 (25.59)	31 (5.25)
Follow-up intervals				
Days between visits (previous appointment), median	89	95	42	52
Visit Type				
In-person, n (%)	2035 (64.7)	1170 (59.6)	399 (67.2)	466 (79.0)
Telemedicine, n (%)	1112 (35.3)	793 (40.4)	195 (32.8)	124 (21.0)
Laboratory testing				
Blood tests performed, n (%)	1587 (50.4)	909 (46.3)	318 (53.5)	360 (61.0)

Abbreviations: AIH: autoimmune hepatitis; IBD: inflammatory bowel disease; MASLD: metabolic-associated steatotic liver disease; MDR: multi-drug-resistant; n: number of patients.

**Table 2:** Appointment distribution across the full cohort and subgroups, including age, the different clinics, performed testing, and required follow-up.

Appointment Distribution	In-Person Visits	Telemedicine Visits
Full cohort, n (%)	2035 (64.7%)	1112 (35.3%)
Age, median	51	53
Hepatology, n (%)	1170 (59.6%)	793 (40.4%)
Viral hepatitis, n (%)	620 (60.1%)	411 (39.9%)
AIH, n (%)	106 (55.2%)	86 (44.8%)
PBC/PSC, n (%)	161 (64.7%)	88 (35.3%)
MASLD, n (%)	231 (53.3%)	202 (46.7%)
Liver transplant, n (%)	399 (67.2%)	195 (32.8%)
IBD, n (%)	466 (79.0%)	124 (21.0%)
Crohn’s disease, n (%)	231 (78.6%)	63 (21.4%)
Ulcerative colitis, n (%)	221 (78.9%)	59 (21.1%)
Follow-up scheduling		
Required early follow-up, %	12.4	8.7
Emergency visits within 30 days, %	4.0	3.8
Laboratory monitoring		
Tests ordered, %	83.9	3.5
Abnormal results requiring action, %	15.2	14.8

Abbreviations: AIH: autoimmune hepatitis; HCV: hepatitis C virus; IBD: inflammatory bowel disease; MASLD: metabolic-associated steatotic liver disease; PBC/PSC: primary biliary cholangitis/primary sclerosing cholangitis.

**Table 3:** Comparison of visit outcomes.

Outcome Measure	In-Person Visits	Telemedicine Visits	*p*-Value
Overall compliance rate, %	90.5	91.3	0.428
Compliance by specialty			
Hepatology, %	89.3	90.3	0.512
Viral Hepatitis, %	88.9	91.8	0.124
AIH, %	88.7	93.0	0.042 *
PBC/PSC, %	89.4	90.9	0.682
MASLD, %	89.6	90.6	0.724
Liver transplant, %	89.5	97.9	<0.001 *
IBD, %	94.4	87.1	0.024 *
Crohn’s disease, %	95.2	87.3	0.018 *
Ulcerative colitis, %	92.8	86.4	0.032 *

Data are presented as percentages. Statistical comparisons for compliance in % between in-person and telemedicine visits performed using chi-square test. For compliance rates, denominators are total scheduled visits per modality; *p* < 0.05 considered statistically significant. Abbreviations: AIH: autoimmune hepatitis; HCV: hepatitis C virus; IBD: inflammatory bowel disease; MASLD: metabolic-associated steatotic liver disease; PBC/PSC: primary biliary cholangitis/primary sclerosing cholangitis. * Statistically significant: a *p*-value <0.05 was considered statistically significant.

**Table 4:** Predictors of telemedicine vs. in-person visit assignment in uni- and multivariate analyses using binary logistic regression analysis.

Variable	Univariate Analysis		Multivariate Analysis	
	OR (95% CI)	*p*-Value	OR (95% CI)	*p*-Value
Clinic type		<0.01 *		<0.01 *
Hepatology	1.00 (Reference)		1.00 (Reference)	
Liver transplant	0.781 (0.64–0.95)		—	
IBD	0.358 (0.28–0.45)		—	
Visit characteristics				
First visit	0.061 (0.03–0.14)	<0.01 *	1.910 (0.35–10.30)	
Days since last visit	1.001 (1.000–1.002)	<0.01 *	1.001 (1.00–1.00)	<0.05 *
Days until next visit	1.003 (1.001–1.004)	<0.01 *	0.999 (0.99–1.00)	
Follow-up type		<0.01 *		<0.01 *
In-person	1.00 (Reference)		1.00 (Reference)	
Telephone	1.406 (1.11–1.79)		—	
Emergency/inpatient	0.591 (0.38–0.92)		—	
Pending	1.330 (1.00–1.78)		—	
Not attended	1.563 (1.04–2.36)		—	
Patient characteristics				
Age at visit	1.009 (1.00–1.01)	<0.01 *	1.002 (0.99–1.01)	
Main diagnosis				
Other	1.00 (Reference)		1.00 (Reference)	
Ulcerative Colitis	0.422 (0.31–0.58)	<0.01 *	0.848 (0.57–1.26)	
Crohn’s Disease	0.422 (0.31–0.57)	<0.01 *	0.649 (0.37–1.15)	
HCV	1.270 (1.01–1.51)	<0.05 *	1.056 (0.80–1.39)	
AIH	1.591 (1.17–2.17)	<0.01 *	1.254 (0.90–1.75)	
MASLD	1.737 (1.40–2.16)	<0.01 *	1.333 (1.05–1.70)	<0.05 *
HBV/HDV coinfection	0.370 (0.19–0.71)	<0.01 *	0.380 (0.19–0.75)	<0.01 *
Post-transplant	1.281 (1.00–1.64)	<0.05 *	1.622 (1.11–2.36)	<0.05 *
Care requirements				
Required endoscopy during study period	0.464 (0.34–0.63)	<0.01 *	0.572 (0.41–0.81)	<0.01 *
Specialist vs. resident care	0.818 (0.70–0.96)	<0.05 *	1.038 (0.85–1.27)	

Odds ratios >1 indicate association with telemedicine visits and <1 with in-person visits. Model adjusted for clinic type, visit timing, follow-up type, age, underlying disease, and procedure requirements. Abbreviations: AIH: autoimmune hepatitis; HCV: hepatitis C virus; IBD: inflammatory bowel disease; MASLD: metabolic-associated steatotic liver disease; Ref.: reference category. * Statistically significant: a *p*-value < 0.05 was considered statistically significant.

## Data Availability

The data that support the findings of this study are available upon request from the corresponding author. The data are not publicly available due to privacy or ethical restrictions.

## Abbreviations

The following abbreviations are used in this manuscript:

AIH	Autoimmune hepatitis
CI	Confidence interval
COVID-19	Coronavirus disease 2019
HBV	Hepatitis B virus
HDV	Hepatitis D virus
IBD	Liver transplant
INR	International normalized ratio
LTX	Liver transplant
MASLD	Metabolic-associated steatotic liver disease
MDR	Multidrug-resistant organisms
MELD	Model for End-Stage Liver Disease
OR	Odds ratio
ORBIS	Hospital’s electronic medical record system, as mentioned
PBC	Primary biliary cholangitis
PSC	Primary sclerosing cholangitis
